# Dysregulation of BDNF/TrkB signaling mediated by NMDAR/Ca^2+^/calpain might contribute to postoperative cognitive dysfunction in aging mice

**DOI:** 10.1186/s12974-019-1695-x

**Published:** 2020-01-16

**Authors:** Li-Li Qiu, Wei Pan, Dan Luo, Guang-Fen Zhang, Zhi-Qiang Zhou, Xiao-Yun Sun, Jian-Jun Yang, Mu-Huo Ji

**Affiliations:** 1 Department of Anesthesiology, Zhongda Hospital, School of Medicine, Southeast University, No. 87 Dingjiaqiao Road, Nanjing, 210009 China; 2 Department of Anesthesiology, Second Affiliated Hospital, School of Medicine, Zhejiang University, Hangzhou, China; 3 Department of Anesthesiology, Jinling Hospital, School of Medicine, Nanjing University, Nanjing, China; 4 Department of Anesthesiology, First Affiliated Hospital of Zhengzhou University, Zhengzhou, China

**Keywords:** Surgery, Cognitive dysfunction, Neuroinflammatioin, NMDAR, Calpain, BDNF, TrkB

## Abstract

**Background:**

Postoperative cognitive decline (POCD) is a recognized clinical phenomenon characterized by cognitive impairments in patients following anesthesia and surgery, yet its underlying mechanism remains unclear. Brain-derived neurotrophic factor (BDNF) plays an important role in neuronal plasticity, learning, and memory via activation of TrkB-full length (TrkB-FL) receptors. It has been reported that an abnormal truncation of TrkB mediated by calpain results in dysregulation of BDNF/TrkB signaling and is associated with cognitive impairments in several neurodegenerative disorders. Calpains are Ca^2+^-dependent proteases, and overactivation of calpain is linked to neuronal death. Since one source of intracellular Ca^2+^ is N-methyl-d-aspartate receptors (NMDARs) related and the function of NMDARs can be regulated by neuroinflammation, we therefore hypothesized that dysregulation of BDNF/TrkB signaling mediated by NMDAR/Ca^2+^/calpain might be involved in the pathogenesis of POCD.

**Methods:**

In the present study, 16-month-old C57BL/6 mice were subjected to exploratory laparotomy with isoflurane anesthesia to establish the POCD animal model. For the interventional study, mice were treated with either NMDAR antagonist memantine or calpain inhibitor MDL-28170. Behavioral tests were performed by open field, Y maze, and fear conditioning tests from 5 to 8 days post-surgery. The levels of Iba-1, GFAP, interleukin-1β (IL-1β), IL-6, tumor necrosis factor-α (TNF-α), NMDARs, calpain, BDNF, TrkB, bax, bcl-2, caspase-3, and dendritic spine density were determined in the hippocampus.

**Results:**

Anesthesia and surgery-induced neuroinflammation overactivated NMDARs and then triggered overactivation of calpain, which subsequently led to the truncation of TrkB-FL, BDNF/TrkB signaling dysregulation, dendritic spine loss, and cell apoptosis, contributing to cognitive impairments in aging mice. These abnormities were prevented by memantine or MDL-28170 treatment.

**Conclusion:**

Collectively, our study supports the notion that NMDAR/Ca2+/calpain is mechanistically involved in anesthesia and surgery-induced BDNF/TrkB signaling disruption and cognitive impairments in aging mice, which provides one possible therapeutic target for POCD.

## Background

Postoperative cognitive decline (POCD) is a recognized clinical phenomenon characterized by cognitive impairments in patients after anesthesia and surgery, especially in the elderly [1]. POCD receives increasing attention because it negatively affects cognitive domains such as memory, attention, and concentration, which are associated with a prolonged hospitalization, a reduced quality of life, and an increased morbidity and mortality [2, 3]. However, its pathophysiology remains unknown.

Brain-derived neurotrophic factor (BDNF) is a neurotrophin widely expressed in the central nervous system, which plays a critical role in neuronal survival and differentiation, and synaptic plasticity through activation of its full-length receptor (TrkB-FL) [4, 5]. Dysregulation of BDNF/TrkB signaling contributes to many pathological processes, including traumatic brain injury [6, 7], brain ischemia [8, 9], and neurodegenerative diseases [10, 11]. However, truncated isoforms of TrkB receptors (TrkB-TC) act as negative modulators of TrkB-FL receptors [12, 13], and alterations in TrkB-TC:TrkB-FL ratio are thought to cause and/or reflect dysregulation of BDNF/TrkB signaling [8, 14]. In an in vitro study, excitotoxic stimulation of cultured rat hippocampal neurons with glutamate downregulated TrkB-FL while upregulated TrkB-TC receptors, which results in dysregulation of BDNF/TrkB signaling [14]. In our previous study, we have showed that decreased expression of BDNF is involved in the pathogenesis of POCD [15]. However, whether TrkB-TC also plays a mechanistic role in POCD remains unclear.

Calpains are intracellular Ca^2+^-dependent cysteine proteases that play a physiologic role by the cleavage of several substrates, including the neurotrophin receptor TrkB [11], cytoskeletal proteins, and membrane receptors [16]. A calpain-dependent truncated form of TrkB-FL has been reported to participate in neurodegenerative diseases, such as AD [11] and epilepsy [17]. The overactivation of calpain could lead to changes in hippocampal structure and function [18] and also be linked to neuronal death [19]. Calpain is overactivated by increased Ca^2+^ concentrations and one source of intracellular Ca^2+^ is NMDARs related. Importantly, one recent study showed that amyloid-β peptide (Aβ) induced the overactivation of NMDARs and calpain, and then triggered the formation of a truncated isoform (TrkB-T′) and an intracellular domain (ICD) fragment, and ultimately disrupted BDNF/TrkB signaling, which can be prevented by a NMDAR antagonist memantine [20]. However, it remains unclear whether the overactivation of NMDARs and a calpain-dependent truncated form of TrkB-FL is involved in the development of POCD.

Inflammation has been proved to be a potential source of reactive oxygen species for inducing NMDARs hypofunction and nonsteroidal anti-inflammatory drugs (NSAIDs) can improve impaired NMDAR-dependent synaptic plasticity and age-related cognitive dysfunction [21]. In addition, accumulating evidence suggests that neuroinflammation plays an initial and central role in anesthesia and surgery-induced cognitive impairments [15, 22, 23]. Upon all these points, we hypothesized that anesthesia and surgery-induced neuroinflammation overactivated NMDARs, and the abnormal activation of NMDARs triggered the overactivation of calpain, which subsequently led to the truncation of TrkB-FL, BDNF/TrkB signaling dysregulation, dendritic spine loss, and cell apoptosis, contributing to cognitive impairments in aging mice.

## Materials and methods

### Animals

**Fig. 1:**
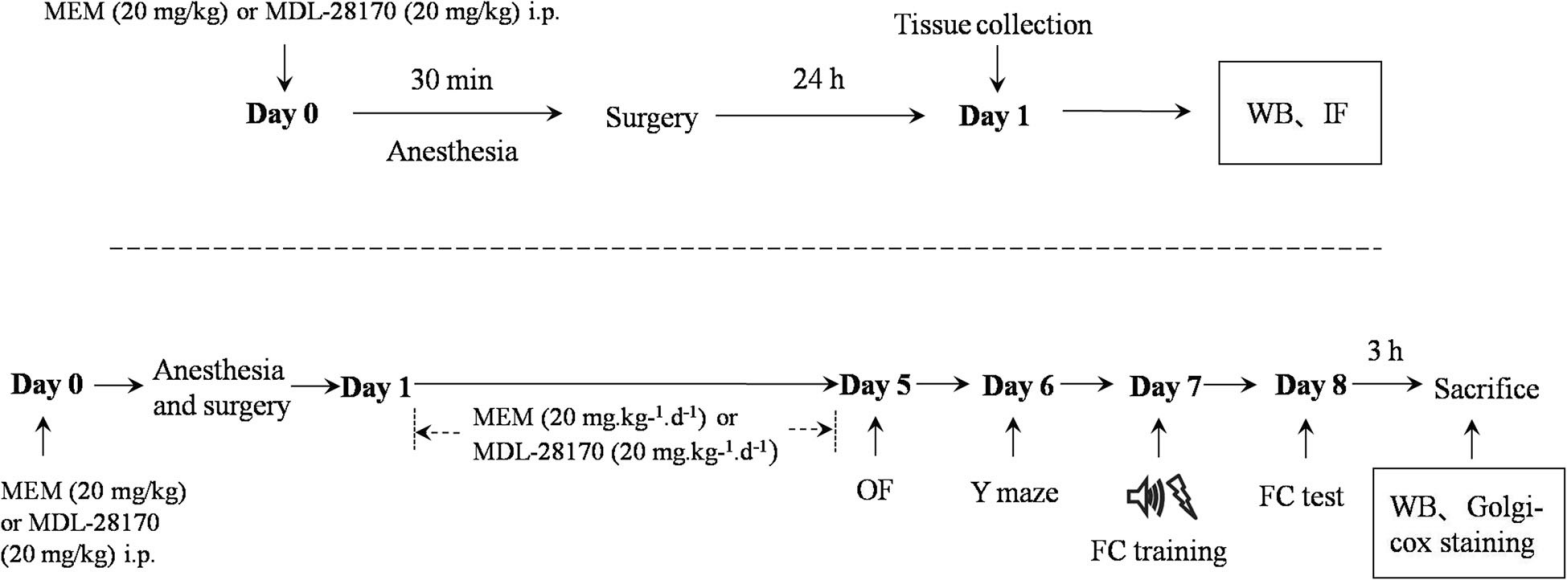
Schematic timeline of the experimental procedure. The mice were treated with MEM (a NMDAR antagonist, 20 mg/kg) or MDL-28170 (a calpain inhibitor, 20 mg/kg) intraperitoneally (i.p.) before anesthesia, and the surgery was performed immediately after 30 min exposure to isoflurane. Twenty-four hours later, the mice were sacrificed for Western blot and immunofluorescence detection. For the behavioral study, mice underwent anesthesia and surgery on day 0. The mice were treated with MEM (20 mg/kg) or MDL-28170 (20 mg/kg) i.p. before anesthesia, and once daily for the subsequent 5 consecutive days. Behavioral tests were performed from 5 to 8 days post-surgery with open field, Y maze, and fear conditioning tests, respectively. Three hours after behavioral tests at 8 days post-surgery, the brains of mice were collected for Western blot and Golgi-Cox staining

One hundred and forty-four 16-month-old male C57BL/6 mice (26–36 g) were obtained from the Animal Center of Southeast University, Nanjing, China. Animals were housed in groups of 3–5 individuals per cage in a standard condition with access to food and water ad libitum in a colony room kept at 19–22 °C and 40–60% humidity, under a 12-h light/dark cycle (light from 07:00 to 19:00). The experiments began after all the animals acclimated to the environment for 2 weeks. The study protocol was approved by the Ethics Committee of Zhongda Hospital, Medical School, Southeast University, and all procedures were performed in accordance with the approved guidelines. The schematic timeline of the experimental procedure is summarized in Fig. 1.

### Animal model

Exploratory laparotomy was performed under isoflurane anesthesia described as our previous studies [15, 23]. Mice were anesthetized in an anesthesia chamber prefilled with 1.5% isoflurane in oxygen. The surgery was performed immediately after 30 min exposure to isoflurane. An abdominal median incision approximately 1 cm was made to allow penetrating the peritoneal cavity. Then the viscera, intestine, and musculature were explored by the operator. Sterile 4-0 chromic gut sutures were used to suture the peritoneal lining and skin. In order to prevent infection, the wound was dressed with polysporin (Pfizer, USA). The surgical procedure was also under isoflurane anesthesia and lasted for 10 min. For the mice that served as controls, neither anesthesia nor surgery was performed.

### Drugs

The NMDAR antagonist memantine (MEM, 20 mg/kg, Sigma, St Louise, MO, USA) [24] was intraperitoneally (i.p.) administered to the mice before anesthesia and then once daily for subsequent 5 consecutive days. The calpain inhibitor, MDL-28170 (20 mg/kg, Sigma, St Louise, MO, USA) [25], was administered to the mice by i.p. before anesthesia and then once daily for the subsequent 5 consecutive days. The selected doses are based on previous studies demonstrating memantine and MDL-28170 confers neuroprotective effects [24, 25].

### Open field

A white opaque plastic chamber (40 cm × 40 cm × 40 cm, XR-XZ301, Shanghai Softmaze Information Technology Co., Ltd., Shanghai, China) was used as the open-field arena. The open-field test was performed at 5 days post-surgery to assess the exploratory locomotor activity. Each mouse was placed in the center of the arena and left to explore it for 5 min while activity was automatically recorded by a video tracking system.

### Y maze

The Y maze is a symmetrical three-arm maze (XR-XY1032; Shanghai Softmaze Information Technology Co., Ltd., Shanghai, China) and is used to evaluate the spontaneous alternation performance at 6 days post-surgery. Each mouse was placed in the center of the Y maze and could explore freely throughout the three different arms of the maze during an 8-min session. The sequence and total number of arms entered were recorded. Arm entry was complete when the hind paws of the mouse had been completely placed in the arm. The alteration was determined from successive consecutive entries to the three different arms on overlapping triads in which all arms were represented. For example, a sequence of entries to the three arms ABC, ACBABACABA, would generate four “successful” alternations, ACB, CBA, BAC, and CAB. Percentage alternation is the number of triads containing entries into all three arms divided by the maximum possible alternations (the total number of arms entered minus 2) × 100. The re-entry into the same arm was not counted for analysis [26].

### Fear conditioning

Mice were trained for fear conditioning at 7 days post-surgery. Each mouse was placed into a conditioning chamber (XR-XC404; Shanghai Softmaze Information Technology Co., Ltd., Shanghai, China) and allowed to explore freely for 3 min. Then a 30-s tone (70 db, 3 kHz) was delivered followed by a 2-s foot shock (0.7 mA). After that, the mouse stayed in the chamber for another 30 s and then returned to the home cage. Contextual fear conditioning (a hippocampus-dependent task) was assessed 24 h after training. For the contextual fear conditioning, each mouse was placed back into the same chamber in which they were explored for 5 min without tone or foot shock and scored for the freezing behavior. Freezing behavior was defined as the absence of all visible movement except for respiration. Given that no difference was observed in the auditory-cued fear test (a hippocampus-independent task) between control and surgery group in our previous studies [15, 23], the test was not performed in the present study.

### Western blot

The entire dissected hippocampus was harvested and subjected to Western blot analysis. The samples were lysed as described previously [15, 23]. Protein concentration was determined by BCA protein assay kit (Beyotime, China). Equivalent amounts of proteins per lane were separated on SDS-PAGE gels and then transferred to polyvinylidinene fluoride (PVDF) membranes. Membranes were blocked with 5% skimmed milk in Tris-buffered saline with Tween (TBST) for 1 h at room temperature. And then the membranes were incubated at 4 °C overnight with primary antibodies including rabbit anti-IL-1β (1:500; Abcam, Cambridge, UK), rabbit anti-IL-6 (1:1000; Affinity, Cincinnati, USA), rabbit anti-TNF-α (1:500, Cell Signaling Technology, Danvers, MA, USA), rabbit anti-GluN2A (1:1000; Abcam, Cambridge, UK), rabbit anti-GluN2B (1:1000; Abcam, Cambridge, UK), mouse anti-αIIspectrin (1:200; Santa Cruz Biotechnology, Dallas, TX, USA), mouse anti-BDNF (1:500; Abcam, Cambridge, UK), mouse anti-TrkB (1:300; Santa Cruz Biotechnology, Dallas, TX, USA), rabbit anti-bax (1:200; Santa Cruz Biotechnology, Dallas, TX, USA), mouse anti-bcl-2 (1:300; Santa Cruz Biotechnology, Dallas, TX, USA), rabbit anti-caspase3 (1:1000; Cell Signaling Technology, Danvers, MA, USA), mouse anti-GAPDH (1:5000; ProteinTech group, Chicago, USA), and rabbit anti-β-tubulin (1:3000; ProteinTech group, Chicago, USA). After washing in TBST three times, the membranes were incubated for 1 h at room temperature with goat anti-rabbit and goat anti-mouse IgG-horseradish peroxidase-conjugated secondary antibodies (1:7000, Bioworld Technology, St. Louis Park, MN, USA). The protein bands were detected by enhanced chemiluminescence, exposed onto X-ray film, and quantitated with Image J software (National Institutes of Health, Bethesda, MD, USA).

### Immunofluorescence

Mice were deeply anesthetized with 2% sodium pentobarbital in saline (60 mg/kg, i.p*.*; Sigma, St Louise, MO, USA) and transcardially perfused with saline, followed by 4% paraformaldehyde (PFA) in phosphate-buffered saline (PBS; pH 7.4). Brains were removed and postfixed in 4% PFA for 2 h and dehydrated in 30% sucrose at 4 °C overnight and then embedded in O.C.T. compound. The brains were cut coronally into 10-μm-thick sections from bregma − 1.70 to − 2.30 by a freezing microtome and mounted on glass slides. The sections were blocked with 1% bovine serum albumin (BSA) for 1 h at room temperature. And then the sections were incubated with primary antibodies: rabbit anti-Iba1 (1:500, Wako Pure Chemical Industries, Osaka, Japan) and rabbit anti-GFAP (1:1000, Sigma, St Louise, MO, USA) in 1% BSA at 4 °C overnight. Sections were washed with PBS three times and incubated with goat anti-rabbit IgG-FITC (1:600; Bioworld Technology, St. Louis Park, MN, USA) and goat anti-rabbit IgG-Cy3 (1:600; Bioworld Technology, St. Louis Park, MN, USA) for 1 h at room temperature. After washing out the secondary antibodies, sections were incubated with 4′,6-diamidino-2-pheny-lindole (DAPI) for nuclear staining. Fluorescent images were captured by a confocal microscope (Olympus, FV1000, Japan). Six sections of the hippocampus per mouse were analyzed by ImageJ (National Institutes of Health, Bethesda, MD, USA) for immunofluorescence analysis. Three non-overlapping fields of each section in the hippocampal Cornu Ammonis 1 (CA1) area was randomly acquired by a counting frame size of 0.4 mm^2^. Positively stained areas were defined that the number of pixels per image with intensity in which was above a predetermined threshold level. The immunoreactivity of a protein was quantified by percentage area with positive staining to the total area of the imaged field. All quantitative analyses were performed by an experimenter blinded to the group of each sample.

### Golgi-Cox staining

The brains of mice were processed at 8 days post-surgery for Golgi-Cox staining [27] using a Golgi Stain Kit (#PK401, FD NeuroTechnologies, Columbia, MD, USA). Briefly, mice were deeply anesthetized by sodium pentobarbital in saline (60 mg/kg, i.p.; Sigma, St Louise, MO, USA) and rapidly sacrificed. The brains were removed as quickly as possible; rinsed in double-distilled water; immersed in impregnation solution, which was a mixture of solutions A and B; and stored in the dark at room temperature (22–25 °C) for 3 weeks. Next, the brains were transferred into solution C and stored for 7 days. Finally, the brains were sliced at a thickness of 100 μm with oscillating tissue slicers, stained and then mounted on gelatin-coated slides. After alcohol dehydration, the tissue sections were cleared in xylene and coverslipped. The hippocampal neurons were captured by an EVOS FL auto microscope (Life technology) under Z-stack mode (× 20 object) for dendritic analysis. The dendrites from hippocampal neurons in CA1 region were captured with a confocal microscope (× 100 oil objective). Dendrite branches were traced by the NeuronJ plugin in ImageJ software, and the dendritic length was calculated. Sholl analysis was applied to measure the dendritic intersections in concentric circles per 20 μm from the cell soma. Dendritic spine density was detected along CA1 secondary dendrites starting from their point of origin on the primary dendrite, and the counting was performed by an experimenter blinded to the group of each sample.

### Statistical analysis

Statistical analyses were analyzed by the GraphPad Prism version 8.0 statistical package (Graphpad Software, Inc.). Data are presented as mean ± S.E.M. Differences between groups were assessed with one-way ANOVA followed by post hoc Tukey multiple comparisons. The dendritic intersections were analyzed by repeated-measures ANOVA followed by post hoc Tukey multiple comparisons. A significant difference was considered as *p* < 0.05.

## Results

### Inhibition of NMDAR attenuated the activation of microglia and astrocytes and proinflammatory cytokines after anesthesia and surgery

**Fig. 2:**
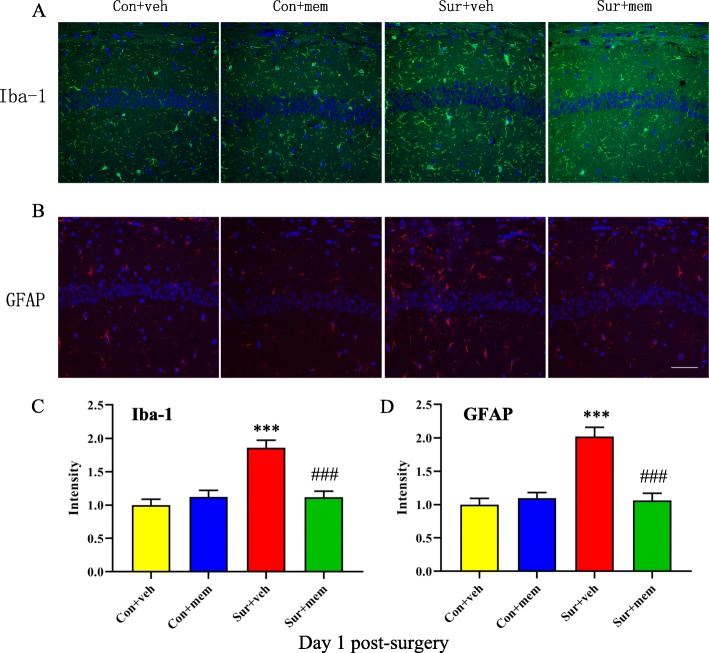
Anesthesia and surgery-induced activation of microglia and astrocytes in the hippocampus was attenuated by MEM treatment on day 1 post-surgery. **a** Representative images of Iba-1 (a marker of microglia) in the hippocampus. **b** Representative images of GFAP (a marker of astrocytes) in the hippocampus. **c** Quantification of Iba-1 fluorescence. **d** Quantification of GFAP fluorescence. Data are presented as the mean ± SEM (*n* = 6). *p < 0.05 compared to the con + veh group (**p < 0.01, ***p < 0.001), ^#^*p <* 0.05 compared to the sur + veh group (^##^*p* < 0.01, ^###^*p* < 0.001). DAPI staining is shown in blue. Scale bar = 50 μm

We performed immunostaining by using antibodies of Iba1 and GFAP on day 1 post-surgery, respectively. Compared with control + vehicle (con + veh) group, the intensity of Iba1 [*F*(3, 20) = 16.34, *p* < 0.0001; Fig. 2a, c] and GFAP [*F*(3, 20) = 20.06, *p* < 0.0001; Fig. 2b, d] was significantly increased in the hippocampus in surgery + vehicle (sur + veh) group. Notably, memantine treatment could attenuate anesthesia and surgery-induced activation of microglia and astrocytes.

**Fig. 3:**
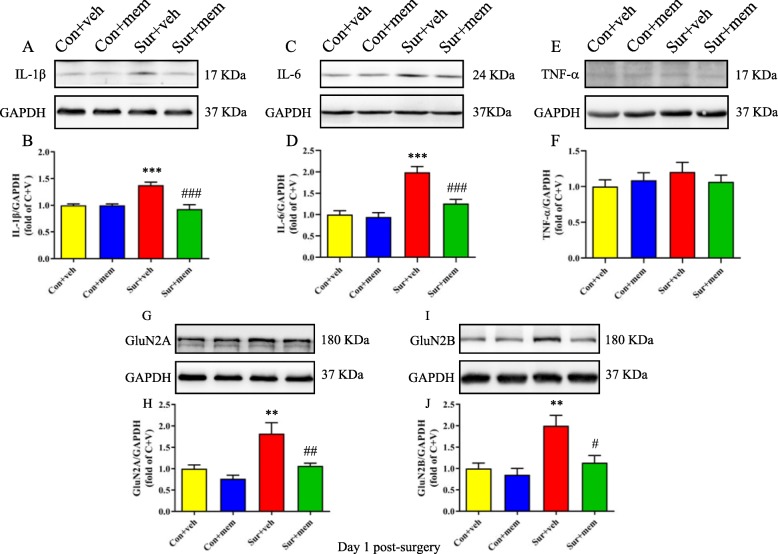
Increased hippocampal levels of IL-1β, IL-6, and NMDAR subunits after anesthesia and surgery were attenuated by MEM treatment on day 1 post-surgery. **a** Representative Western blots of IL-1β in the hippocampus. GAPDH was included as a loading control. **b** Quantitative analysis of IL-1β levels. **c** Representative Western blots of IL-6 in the hippocampus. GAPDH was included as loading control. **d** Quantitative analysis of IL-6 levels. **e** Representative Western blots of TNF-α in the hippocampus. GAPDH was included as loading control. **f** Quantitative analysis of TNF-α levels. **g** Representative Western blots of GluN2A. GAPDH was included as loading control. **h** Quantitative analysis of GluN2A levels. **i** Representative Western blots of GluN2B. GAPDH was included as loading control. **j** Quantitative analysis of GluN2B levels. Data are presented as the mean ± SEM (*n* = 6). *p < 0.05 compared to the con + veh group (**p < 0.01, ***p < 0.001), ^#^*p <* 0.05 compared to the sur + veh group (^##^*p* < 0.01, ^###^*p* < 0.001)

**Fig. 4:**
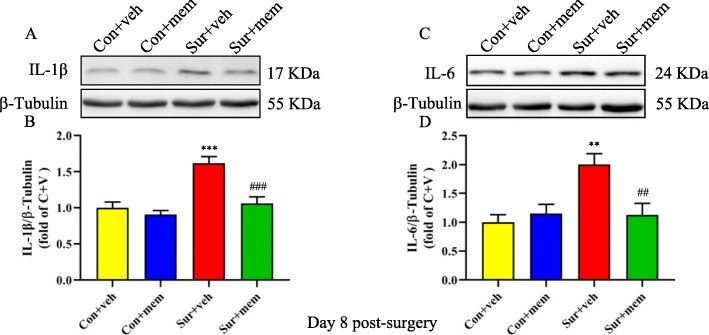
Increased hippocampal levels of IL-1β and IL-6 after anesthesia and surgery were detected on day 8 post-surgery, which were attenuated by MEM treatment. **a** Representative Western blots of IL-1β in the hippocampus. β-tubulin was included as loading control. **b** Quantitative analysis of IL-1β levels. **c** Representative Western blots of IL-6 in the hippocampus. β-tubulin was included as loading control. **d** Quantitative analysis of IL-6 levels. Data are presented as the mean ± SEM (*n* = 6). *p < 0.05 compared to the con + veh group (**p < 0.01, ***p < 0.001), #*p <* 0.05 compared to the sur + veh group (^##^*p* < 0.01, ^###^*p* < 0.001)

Next, we detected the levels of proinflammatory cytokines on days 1 and 8 post-surgery. On day 1 post-surgery, the IL-1β [*F*(3, 20) = 14.55, *p* < 0.0001; Fig. 3a, b] and IL-6 [*F*(3, 20) = 19.50, *p* < 0.0001; Fig. 3c, d] levels were significantly increased in the sur + veh group, which was attenuated in the surgery + MEM (sur + MEM) group. On day 8 post-surgery, we also showed that the increased levels of IL-1β [*F*(3, 20) = 16.02, *p* < 0.0001; Fig. 4a, b] and IL-6 [*F*(3, 20) = 7.250, *p* = 0.0018; Fig. 4c, d] induced by anesthesia and surgery, which were reversed by memantine treatment. But there were no significant differences of tumor necrosis factor-α (TNF-α) among the groups [*F*(3, 20) = 0.6109, *p* = 0.6158; Fig. 3e, f]. The levels of GluN2A and GluN2B of the hippocampus were also measured using Western blot. GluN2A [*F*(3, 20) = 10.13, *p* = 0.0003; Fig. 3g, h] and GluN2B [*F*(3, 20) = 8.462, *p* = 0.0008; Fig. 3i, j] levels were significantly increased in the sur + veh group, which were reversed by memantine treatment.

### Inhibition of NMDAR limited the activation of calpain and TrkB-FL cleavage after anesthesia and surgery

**Fig. 5:**
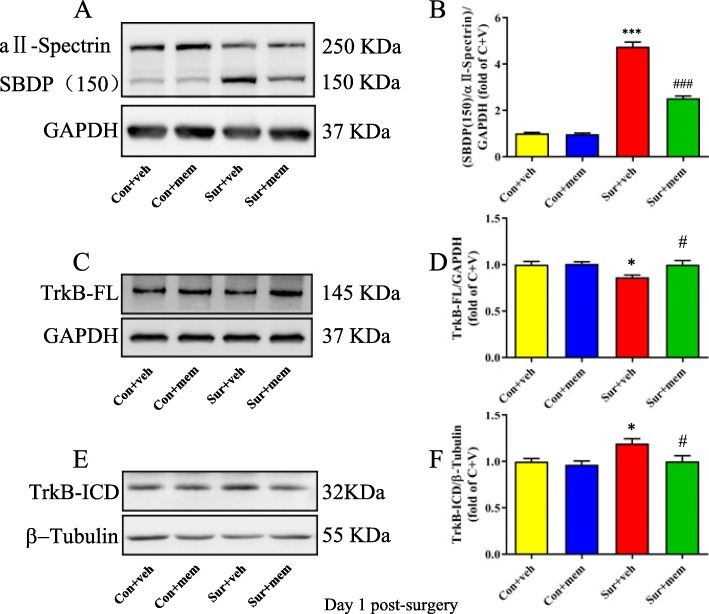
MEM treatment reduced the cleavage of TrkB by modulating the overactivation of calpain on day 1 post-surgery. **a** Representative Western blots of SBDP in the hippocampus. GAPDH was included as loading control. **b** Quantitative analysis of the ratio of the SBDP to αII-spectrin. **c** Representative Western blots of TrkB-FL. GAPDH was included as loading control. **d** Quantitative analysis of TrkB-FL levels. **e** Representative Western blots of TrkB-ICD. β-tubulin was included as loading control. **f** Quantitative analysis of TrkB-ICD levels. Data are presented as the mean ± SEM (*n* = 6). *p < 0.05 compared to the con + veh group (**p < 0.01, ***p < 0.001), #*p <* 0.05 compared to the sur + veh group (^##^*p* < 0.01, ^###^*p* < 0.001)

To evaluate whether NMDAR inhibition by memantine could affect anesthesia and surgery-induced activation of calpain, we detected αII-spectrin levels and the formation of calpain-specific spectrin breakdown products (SBDPs). αII-spectrin is a major substrate for calpain and caspase-3 proteases. The activation of calpain could result in the cleavage of αII-spectrin and then produce breakdown products with distinct molecular weights. The expected molecular weight of αII-spectrin is 250 kDa, whereas the SBDP is 150 kDa. Our data showed that the levels of SBDPs were significantly increased, which was accompanied by decreased levels of αII-spectrin in the sur + veh group, suggesting the activation of calpain. However, this increase was ameliorated by memantine treatment [*F*(3, 20) = 234.5, *p* < 0.0001; Fig. 5a, b]. Next, we evaluated the effects of memantine on TrkB levels after anesthesia and surgery. We observed that anesthesia and surgery induced a marked decrease in TrkB-FL [*F*(3, 20) = 4.599, *p* = 0.0132; Fig. 5c, d] and a significant increase in TrkB-ICD levels [*F*(3, 20) = 4.854, *p* = 0.0107; Fig. 5e, f]. However, memantine treatment reversed these alterations (Fig. 5).

### Inhibition of NMDAR or calpain reversed BDNF/TrkB signaling disruption and decreased apoptosis after anesthesia and surgery

**Fig. 6:**
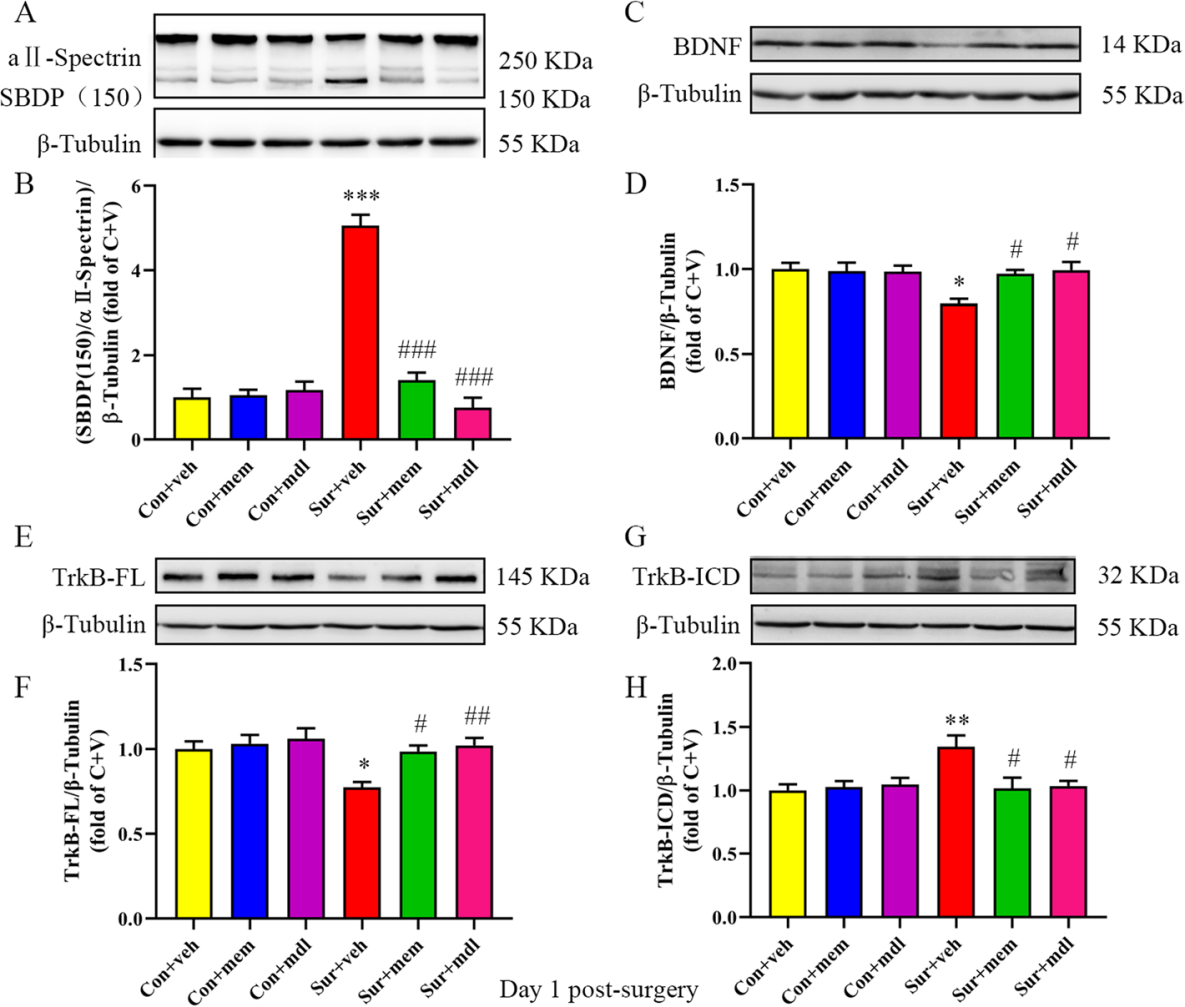
Inhibition of NMDAR or calpain restored BDNF/TrkB signaling disruption on day 1 post-surgery. **a** Representative Western blots of SBDP in the hippocampus. β-tubulin was included as loading control. **b** Quantitative analysis of the ratio of the SBDP to αII-spectrin. **c** Representative Western blots of BDNF in the hippocampus. β-tubulin was included as loading control. **d** Quantitative analysis of BDNF levels. **e** Representative Western blots of TrkB-FL. β-tubulin was included as loading control. **f** Quantitative analysis of TrkB-FL levels. **g** Representative Western blots of TrkB-ICD. β-tubulin was included as loading control. **h** Quantitative analysis of TrkB-ICD levels. Data are presented as the mean ± SEM (*n* = 6). *p < 0.05 compared to the con + veh group (**p < 0.01, ***p < 0.001), ^#^*p <* 0.05 compared to the sur + veh group (^##^*p* < 0.01, ^###^*p* < 0.001)

To determine whether TrkB is truncated by calpain activation, we administrated MDL-28170, an inhibitor of calpain. The levels of SBDPs were significantly increased and the levels of αII-spectrin were decreased after anesthesia and surgery, which were reversed by memantine or MDL-28170 treatment [*F*(5, 30) = 64.15, *p* < 0.0001; Fig. 6a, b]. BDNF levels were significantly decreased in the sur + veh group, whereas memantine or MDL-28170 treatment reversed the decreased BDNF levels [*F*(5, 30) = 4.064, *p* = 0.0062; Fig. 6c, d]. We also showed that anesthesia and surgery induced a marked decrease in TrkB-FL levels [*F*(5, 30) = 4.958, *p* = 0.0020; Fig. 6e, f] and a significant increase in TrkB-ICD levels [*F*(5, 30) = 4.325, *p* = 0.0044; Fig. 6g, h]. Again, memantine or MDL-28170 treatment reversed these alterations (Fig. 6).

**Fig. 7:**
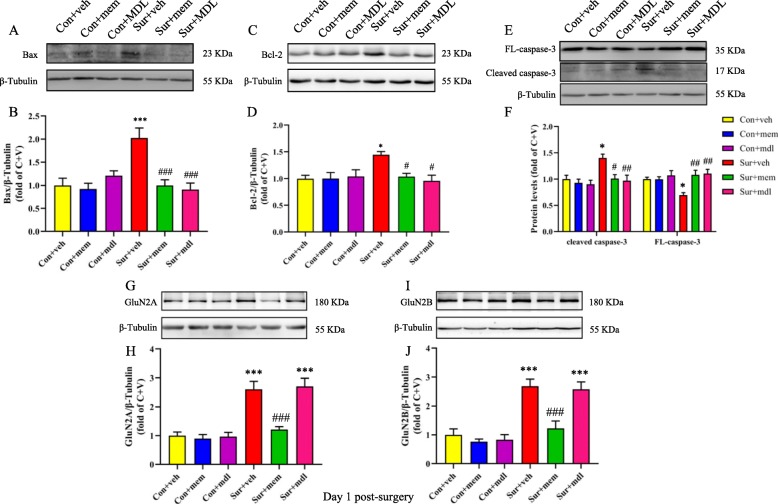
Increased levels of markers associated with cell apoptosis were attenuated by MEM or MDL-28170 treatment on day 1 post-surgery. **a** Representative Western blots of bax in the hippocampus. β-tubulin was included as loading control. **b** Quantitative analysis of bax. **c** Representative Western blots of bcl-2. β-tubulin was included as loading control. **d** Quantitative analysis of bcl-2 levels. **e** Representative Western blots of FL-caspase-3 and cleaved caspase-3 in the hippocampus. β-tubulin was included as loading control. **f** Quantitative analysis of cleaved caspase-3 and FL-caspase-3 levels. **g** Representative western-blots of GluN2A. β-tubulin was included as loading control. **h** Quantitative analysis of GluN2A levels. **i** Representative Western blots of GluN2B. β-tubulin was included as loading control. **j** Quantitative analysis of GluN2B levels. Data are presented as the mean ± SEM (*n* = 6). *p < 0.05 compared to the con+ veh group (**p < 0.01, ***P < 0.001), #*p <* 0.05 compared to the sur + veh group (^##^*p* < 0.01, ^###^*p* < 0.001)

The presence of calpain-cleaved fragments occurs early in neural cell pathology and may be indicative of necrotic and excitotoxic neuronal injury and death. As reported previously, cell apoptosis plays an important role in POCD. In the current study, we showed that the levels of bax [*F*(5, 30) = 8.311, *p* < 0.0001; Fig. 7a, b], bcl-2 [*F*(5, 30) = 3.739, *p* = 0.0095; Fig. 7c, d], and cleaved caspase-3 [*F*(5, 30) = 5.399, *p* = 0.0012; Fig. 7e, f] were significantly increased, and FL-caspase-3 [*F*(5, 30) = 4.754, *p* = 0.0026; Fig. 7e, f] levels were significantly decreased after anesthesia and surgery, while memantine or MDL-28170 treatment could reverse the above alterations (Fig. 7). We also observed that memantine treatment could reduce increased GluN2A [*F*(5, 30) = 19.98, *p* < 0.0001; Fig. 7g, h] and GluN2B [*F*(5, 30) = 17.00, *p* < 0.0001; Fig. 7i, j] levels after anesthesia and surgery. However, MDL-28170 treatment did not change NMDARs levels (Fig. 7).

### Inhibition of NMDAR or calpain attenuated anesthesia and surgery-induced hippocampal dendritic spine loss

**Fig. 8:**
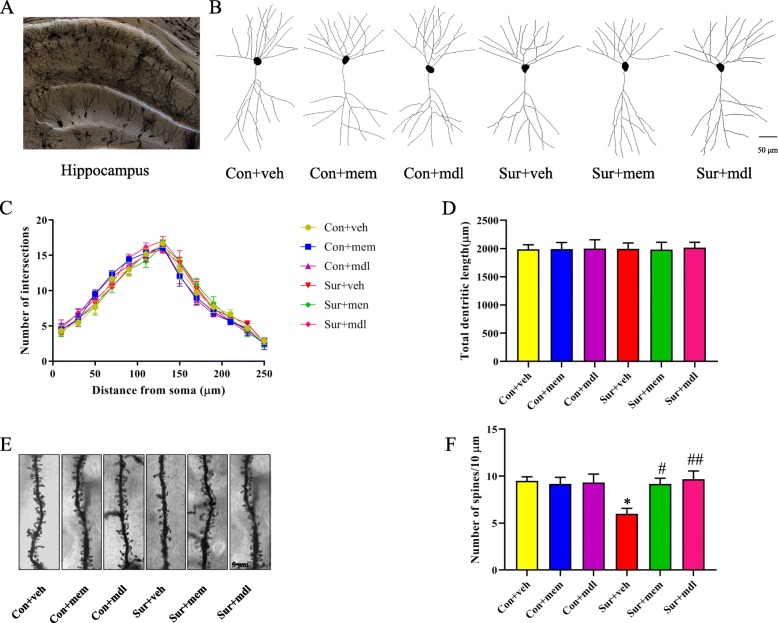
Inhibition of NMDAR or calpain attenuated hippocampal dendritic density loss after anesthesia and surgery. **a** A hippocampal profile image of Golgi-Cox staining. **b** Representative camera tracings of hippocampal CA1 neurons of the six groups. **c** Quantitation of the dendritic intersections of the six groups. **d** Quantitation of the total dendritic length of the six groups. **e** Representative dendritic spine density of hippocampal CA1 neurons of the six groups. **f** Quantitation of the dendritic spine density of the six groups. Data are presented as the mean ± SEM (*n* = 6). *p < 0.05 compared to the con + veh group (**p < 0.01, ***p < 0.001), ^#^*p <* 0.05 compared to the sur + veh group (^##^*p* < 0.01, ^###^*p* < 0.001)

We used Sholl analysis to assess dendritic branching and spine density in the CA1 region in the hippocampus. There was no significant difference in the total number of dendritic intersections [*F*(5, 30) = 0.1977, *p* = 0.9609; Fig. 8c] and total dendritic length [*F*(5, 30) = 0.009950, *p* > 0.9999; Fig. 8d] among the six groups. However, the dendritic spine density was significantly reduced after anesthesia and surgery, while memantine or MDL-28170 treatment attenuated anesthesia and surgery-induced dendritic spine loss [*F*(5, 30) = 3.949, *p* = 0.0072; Fig. 8e, f].

### Inhibition of NMDAR or calpain attenuated cognitive impairments after anesthesia and surgery

**Fig. 9:**
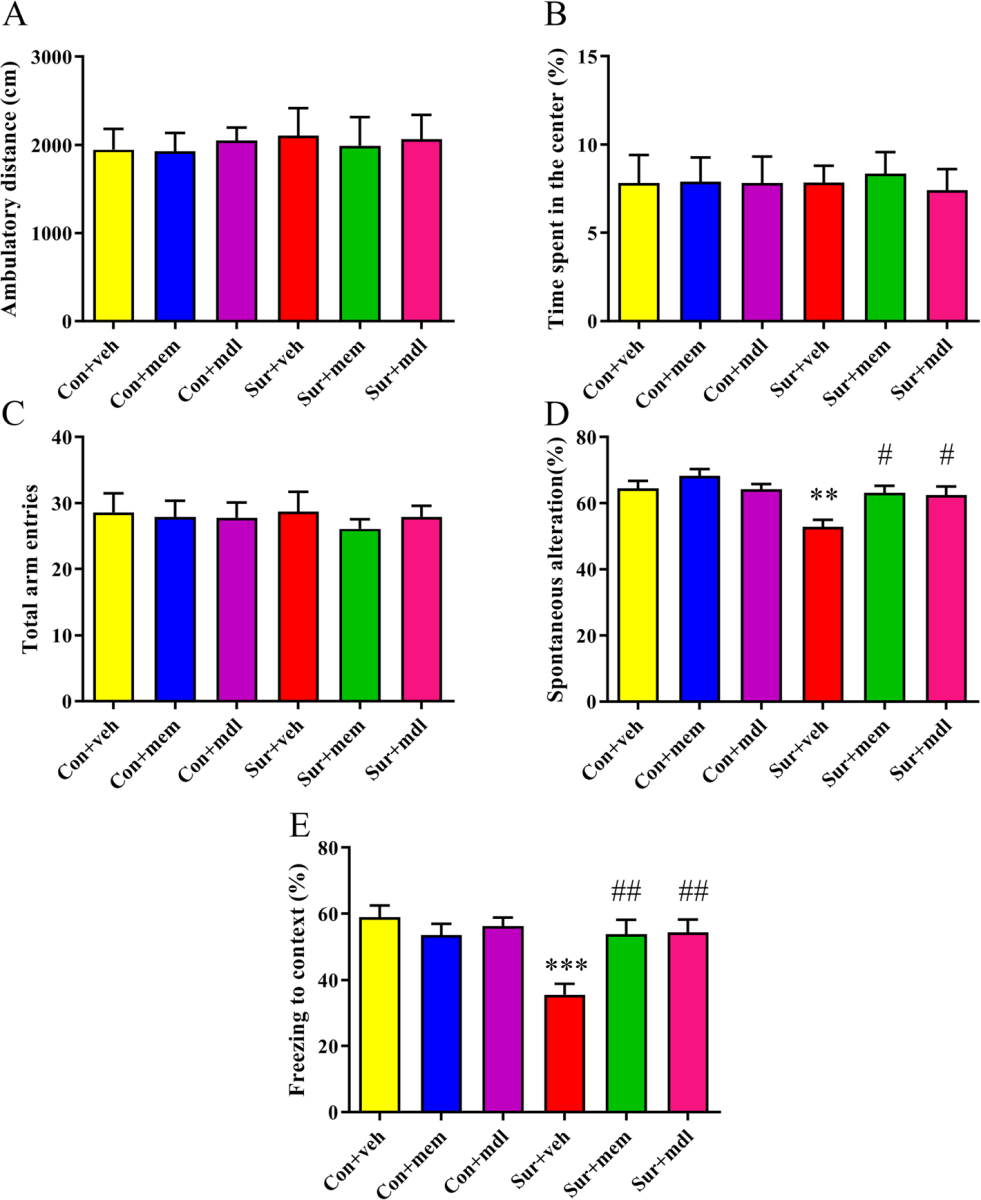
Anesthesia and surgery-induced cognitive impairments were attenuated by MEM or MDL-28170 treatment. **a**, **b** There was no significant difference at ambulatory distance and time spent in the center in the open field test among the six groups at 5 days post-surgery. **c** There was no significant difference of the total arm entries in the Y maze test. **d** The mice in the sur + veh group displayed lesser spontaneous alteration than the mice in the con + veh group, which was reversed by MEM or MDL-28170 treatment. **e** The freezing time to context was significantly decreased after anesthesia and surgery, while MEM or MDL-28170 treatment evidently increased the freezing time. Data are presented as the mean ± SEM (*n* = 10). *p < 0.05 compared to the con+ veh group (**p < 0.01, ***p < 0.001), ^#^*p <* 0.05 compared to the sur + veh group (^##^*p* < 0.01, ^###^*p* < 0.001)

The open field was used to evaluate the locomotor activity and exploratory behavior. During the 5-min test session, there was no significant difference in the total distance [*F*(5, 54) = 0.07516, *p* = 0.9957; Fig. 9a] and the time spent in the center of the arena [*F*(5, 54) = 0.05177, *p* = 0.9982; Fig. 9b] among the six groups at 5 days post-surgery. At 6 days post-surgery, mice were tested in the spontaneous alternation Y-maze paradigm that assesses spatial working memory. There was no significant difference in total arm entries among the six groups [*F*(5, 54) = 0.1552, *p* = 0.9776; Fig. 9c]. The mice in the sur + veh group displayed lesser spontaneous alteration than the mice in con + veh group, which was reversed by memantine or MDL-28170 treatment [*F*(5, 54) = 6.052, *p* = 0.0002; Fig. 9d]. At 8 days post-surgery, the contextual fear conditioning test was used to access the long-term memory. Mice in the sur + veh group displayed significantly decreased freezing time than those in the con + veh group in the contextual fear conditioning test, which was reversed by memantine or MDL-28170 treatment [*F*(5, 54) = 5.489, *p* = 0.0004; Fig. 9e].

## Discussion

**Fig. 10:**
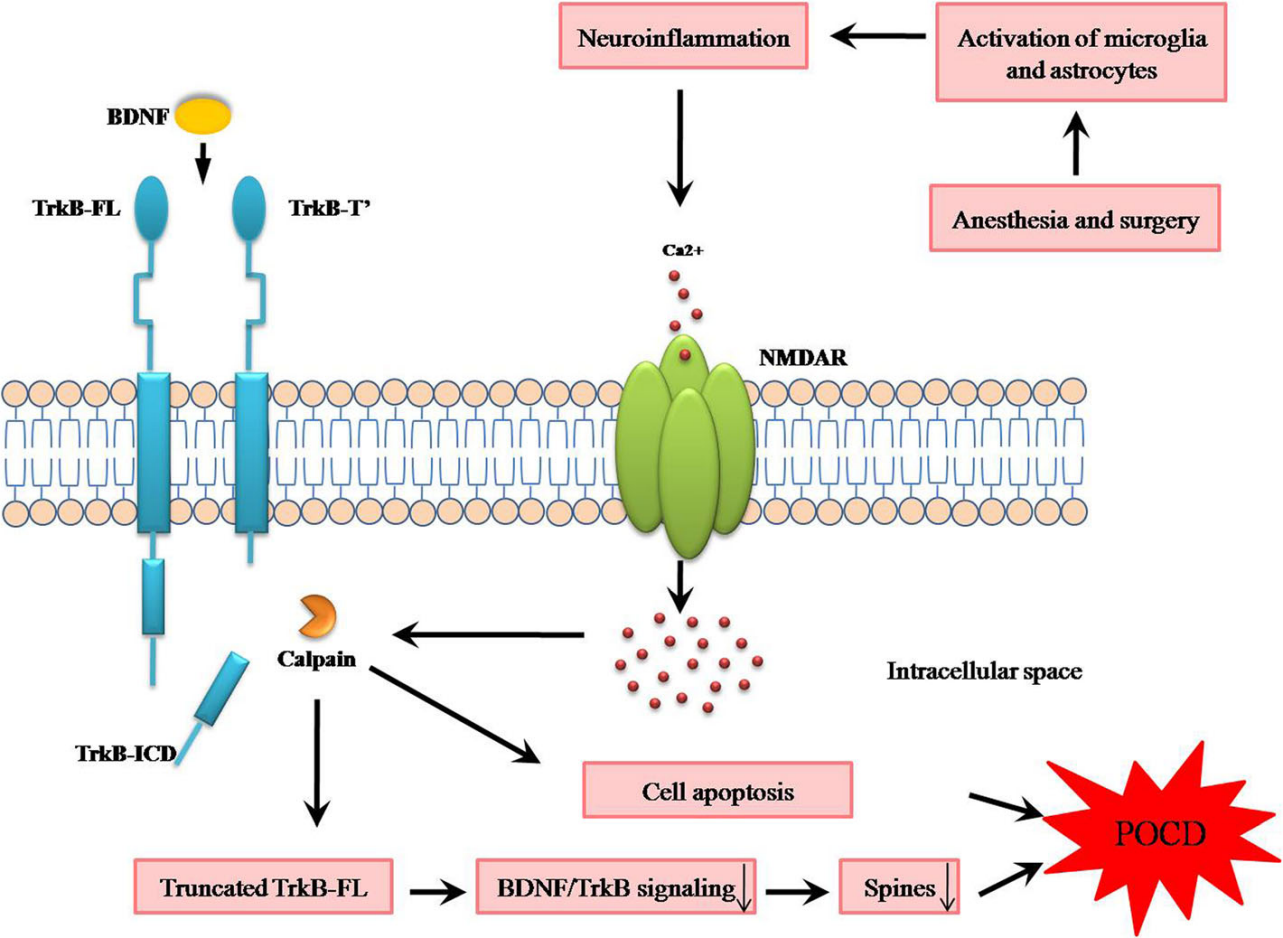
General overview of the main highlights of this study. Anesthesia and surgery-induced neuroinflammation overactivated NMDARs and calpain, which subsequently led to the truncation of TrkB-FL, BDNF/TrkB signaling dysregulation, dendritic spine loss, and cell apoptosis, contributing to cognitive impairments in aging mice

In the present study, we showed that anesthesia and surgery-induced neuroinflammation overactivated NMDARs, and the abnormal activation of NMDARs triggered the overactivation of calpain, which subsequently led to the truncation of TrkB-FL, BDNF/TrkB signaling dysregulation, dendritic spine loss, and cell apoptosis, contributing to cognitive impairments in aging mice. Of note, NMDARs antagonist memantine or calpain inhibitor MDL-28170 attenuated these abnormalities, suggesting tackling abnormal activation of NMDARs or truncation of TrkB-FL may be a therapeutic strategy for POCD (Fig. 10).

Patients suffering from cognitive impairments after anesthesia and surgery have been recognized for more than 60 years. Recently, a multi-specialty working group recommended “perioperative neurocognitive disorders” (PND) as an overarching term for cognitive impairments diagnosed in the preoperative or postoperative period [28–33]. PND includes cognitive decline diagnosed before operation (described as neurocognitive disorder), postoperative delirium (POD), delayed neurocognitive recovery, and POCD. Since we focused on cognitive performance during the postoperative period, we still used the term of POCD in the current study.

Although various mechanisms have been proposed to be involved in the development of POCD, neuroinflammation is believed to play an initial and central role. In the present study, we showed that IL-1β and IL-6 levels were significantly increased and lasted for 8 days post-surgery. Our results were consistent with the previous studies [15, 22, 34], suggesting that the hippocampus is susceptible to neuroinflammation induced by anesthesia and surgery. However, the mechanism by which neuroinflammation leading to cognitive impairments following anesthesia and surgery remains unclear.

There is accumulating evidence suggesting a strong correlation between neuroinflammation and NMDAR dysfunction, eventually resulting in deficits of synaptic plasticity and cognitive impairments. It has been showed that microglia is activated initially and become a major cellular source of a variety of proinflammatory cytokines in a rat model of chronic neuroinflammation, which has negative effect on long-term potentiation requiring NMDAR activation [35]. In addition, the anti-inflammatory agent indomethacin has been reported to improve cognitive impairments by inhibiting microglia activation and reversing NMDAR dysfunction in aged rats [21]. On the other hand, another study has showed that NMDA-induced retinal excitotoxicity could trigger microglia recruitment and IL-1β production [36]. In our study, we found that anesthesia and surgery induced neuroinflammation and NMDAR overactivation. Although we do not know which signaling works as an initial trigger, we speculate there is a possible cross-talk between neuroinflammation and NMDAR overactivation, leading to cognitive impairments in aging mice.

We previously demonstrated that NMDARs calcium/calmodulin-dependent kinase II pathway was involved in the pathogenesis of POCD [37]. Therefore, the present study tested the hypothesis that NMDARs may be implicated in the overactivation of calpain in POCD. NMDARs are ligand-gated ion channels, which have tetrameric structure composed of GluN1, GluN2A-D, and GluN3A-B subunits, forming di- or triheteromers with participation of GluN1. The most widely expressed NMDARs contain the obligatory subunit GluN1 plus either GluN2A or GluN2B or a mixture of the two. In particular, NR2B is involved in NMDA-mediated excitotoxicity [38], while NR2A is associated with human immunodeficiency virus-mediated neurotoxicity [39]. Here, we showed the levels of GluN2A and GluN2B were significantly increased after anesthesia and surgery. The treatment of memantine, a low-affinity uncompetitive NMDAR antagonist without affecting normal physiological activity, attenuated anesthesia and surgery-induced overactivation of NMDARs and calpain and improved cognitive impairments in aging mice. It has been previously demonstrated that memantine is neuroprotective in various brain diseases, including AD [40], Parkinson’s disease [41], traumatic brain injury [42], and pain-induced cognitive impairments [43], which involves its anti-inflammation, anti-oxidation, anti-apoptosis, and anti-glutamate excitotoxicity properties. In our study, we showed that NMDAR/Ca^2+^/calpain is mechanistically involved in neuroprotective effects of memantine in anesthesia and surgery-induced cognitive impairments in aging mice. In a rat model of postoperative pain-induced cognitive impairments, memantine treatment could improve memory deficits without any effect on NMDAR expression [43]. Interestingly, we showed that memantine downregulated the levels of GluN2A and GluN2B. Indeed, one previous study suggests that memantine inhibited ethanol-induced upregulation of NMDA receptor subunits GluN2A and GluN2B in rat hippocampal neurons [44]. Thus, this downregulation of GluN2A and GluN2B levels by memantine seems to be responsible for its neuroprotective effects in aging mice after anesthesia and surgery.

BDNF plays an important role in neuronal plasticity, learning, and memory through the activation of TrkB-FL receptors [4, 5]. It has been reported that reduced BDNF signaling through TrkB-FL leads to impaired memory [45, 46]. IL-1β has been shown to negatively regulate BDNF-dependent learning and memory in neurodegenerative diseases [47]. In our previous study, we demonstrated that anesthesia and surgery could induce microglial activation, I L-1β release, and BDNF downregulation in the hippocampus and thus resulted in hippocampus-dependent cognitive impairments in aged mice [15]. In support, we showed that microglia and astrocyte-induced neuroinflammation plays a crucial role in the development of POCD. Moreover, alteration in BDNF signaling can result in synaptic dysfunction that is associated with memory deficits observed in AD [48], Parkinson’s disease (PD) [41], stroke [49], and sepsis-associated encephalopathy [50]. In the present study, we showed that anesthesia and surgery induced decreased levels of BDNF, which could be reversed by memantine and MDL-28170. The results are consistent with a previous study in PD animal model [41]. Therefore, we speculate that neuroinflammation and overactivated calcium signaling pathway may contribute to the downregulation of BDNF levels after anesthesia and surgery in aging mice. However, it remains unclear whether the alteration of TrkB is involved in the development of POCD.

TrkB is expressed as a full-length, catalytically active isoform (TrkB-FL), as well as several alternatively spliced truncated isoforms lacking the intracellular kinase domain, including TrkB.T1, TrkB.T2, and TrkB.T-Shc [51, 52]. Both TrkB-FL and TrkB-TC are widely expressed throughout the adult mammalian CNS [53]. Abnormal levels of TrkB-TC have been reported in several neurodegenerative disorders, such as AD [11], Down syndrome [12], and amyotrophic lateral sclerosis [54]. It has been reported that overexpression of TrkB-FL improves spatial memory in mice [55], whereas overexpression of TrkB.T1 slightly impairs it [46]. In a mouse model of Down syndrome, the accelerated death of hippocampal neurons is not rescued by exogenous BDNF delivery [12] but instead by restoring the physiological levels of TrkB.T1 [56]. In a mouse model of spinal cord injury, the increased levels of TrkB.T1 contribute to locomotor dysfunction and neuropathic pain [57]. In the present study, we showed that expression of TrkB-FL was significantly decreased after anesthesia and surgery, whereas TrkB-ICD was significantly increased. Increasing evidence has showed that excitotoxicity is associated with the downregulation of TrkB-FL and upregulation of TrkB-TC expression [14]. The abnormal activation of calpain has been shown to associate with excitotoxicity and mediates neuronal injury by cleavage on TrkB-FL receptor in stroke and ischemic neurodegeneration [58]. It has also been demonstrated that Aβ induced a calpain-mediated cleavage on TrkB-FL receptors, producing a new truncated TrkB receptor (TrkB-T′) and a 32-kDa intracellular fragment (TrkB-ICD), which was also detected in postmortem human brain samples [11]. Besides, the overactivation of calpain has been associated with several neuropsychiatric disorders, including Huntington’s disease [59], Parkinson’s disease [60], and brain trauma [61]. In our study, anesthesia and surgery induced overactivation of calpain and subsequently resulted in increased TrkB-ICD and decreased TrkB-FL levels, which were rescued by calpain inhibitor MDL-28170. This suggests that the strategy targeting at calpain is a promising therapeutic strategy for cognitive impairment after anesthesia and surgery.

There are some limitations in this study. Firstly, we mainly observed relatively short-term cognitive performance by Y maze and fear conditioning tests after anesthesia and surgery. Using Morris water maze to measure spatial cognition are needed in our future studies. Secondly, memantine is a partial antagonist of NMDAR receptor that block the pathological activation of NMDARs, while not interfering with normal synaptic transmission [62]. Clinically, memantine is a commercially available and FDA-approved drug used for the treatment of AD patients. That is the reason why we did not use the selective GluN2A and GluN2B subunit antagonists in this study. In addition, we only detected a 32-kDa intracellular fragment (TrkB-ICD), but not new truncated TrkB receptor, which deserves further study in the future.

## Conclusions

In summary, our study demonstrated that neuroinflammation overactivated NMDARs, at least in part, plays a key role in overactivation of calpain, cleavage of TrkB-FL receptor, BDNF/TrkB signaling dysfunction, dendritic spine loss, cell apoptosis, and consequent cognitive impairments. Hence, identifying viable therapeutic strategies to tackle abnormal activation of NMDARs or calpain may provide effective interventions for POCD.

## Data Availability

The data supporting the findings of this study are presented within the manuscript.

## Abbreviations

Aβ: Amyloid-β
BDNF: Brain-derived neurotrophic factor
BSA: Bovine serum albumin
DAPI: 4′,6-diamidino-2-pheny-lindole
i.p.: Intraperitoneally
ICD: An intracellular domain
IL-1β: Interleukin-1β
MEM: Memantine
NMDAR: N-methyl-d-aspartate receptor
NSAIDs: Nonsteroidal anti-inflammatory drugs
PBS: Phosphate-buffered saline
PFA: Paraformaldehyde
PND: Perioperative neurocognitive disorders
POCD: Postoperative cognitive decline
POD: Postoperative delirium
SBDPs: Calpain-specific spectrin breakdown products
TNF-α: Tumor necrosis factor-α
TrkB-FL: TrkB-full length
TrkB-T′: New truncated TrkB
TrkB-TC: Truncated isoforms of TrkB
